# Broad Spectrum of Mimiviridae Virophage Allows Its Isolation Using a Mimivirus Reporter

**DOI:** 10.1371/journal.pone.0061912

**Published:** 2013-04-15

**Authors:** Morgan Gaia, Isabelle Pagnier, Angélique Campocasso, Ghislain Fournous, Didier Raoult, Bernard La Scola

**Affiliations:** URMITE, UM63, CNRS 7278, IRD 198, Inserm 1095, Aix Marseille Universite, Marseille, France; Hospital for Sick Children, Canada

## Abstract

The giant virus Mimiviridae family includes 3 groups of viruses: group A (includes *Acanthamoeba polyphaga Mimivirus*), group B (includes Moumouvirus) and group C (includes *Megavirus chilensis*). Virophages have been isolated with both group A Mimiviridae (the Mamavirus strain) and the related Cafeteria roenbergensis virus, and they have also been described by bioinformatic analysis of the Phycodnavirus. Here, we found that the first two strains of virophages isolated with group A Mimiviridae can multiply easily in groups B and C and play a role in gene transfer among these virus subgroups. To isolate new virophages and their Mimiviridae host in the environment, we used PCR to identify a sample with a virophage and a group C Mimiviridae that failed to grow on amoeba. Moreover, we showed that virophages reduce the pathogenic effect of Mimivirus (plaque formation), establishing its parasitic role on Mimivirus. We therefore developed a co-culture procedure using *Acanthamoeba polyphaga* and Mimivirus to recover the detected virophage and then sequenced the virophage's genome. We present this technique as a novel approach to isolating virophages. We demonstrated that the newly identified virophages replicate in the viral factories of all three groups of Mimiviridae, suggesting that the spectrum of virophages is not limited to their initial host.

## Introduction

Free-living amoebas are ubiquitous protozoa that feed on microorganisms in their environment through phagocytosis. However, some microorganisms are able to resist digestion by this predator after phagocytosis [1]. Thus, amoebas have been used as a tool for the isolation of digestion-resistant environmental bacteria, such as *Legionella* sp. [2]. This technique allowed the fortuitous discovery of the first giant virus, *Acanthamoeba polyphaga Mimivirus* (APM) [3]. APM is a large icosahedral virus with a 500 nm capsid [4] that is covered with surrounding fibrils and contains a 1.2 Mbp genome [5]. It belongs to the nucleocytoplasmic large DNA virus (NCLDV) family in the recently proposed order Megavirales [6], which also includes Iridoviridae, Phycodnaviridae, Asfarviridae, Ascoviridae and Poxviridae [7]. A second closely related but slightly larger APM was later isolated, and the strain was called Mamavirus [8]. Since the isolation of the first two giant viruses in our laboratory, we have improved the co-culture technique, established a collection of APM and reported 18 isolates [9]. We have established a preliminary phylogenetic tree based on partial polB sequences; the tree shows a repartition into three groups: group A (includes the Mimivirus and Mamavirus), group B (includes the Moumouvirus) and group C (includes the recently described *Megavirus chilensis*) [6].

We isolated a small virus that co-isolates with Mamavirus and infects the Mamavirus virus factory. We named this new virus Sputnik and classified it as the first virophage [8]. This icosahedral, 50 nm virus with an 18 kb DNA genome is deleterious for Mimivirus growth. Later, we identified an isolate (Lentille) in our APM collection that was associated with a new strain of virophage; we named the new virophage strain Sputnik 2 [9]. More recently, another virophage was isolated with *Cafeteria roenbergensis* virus, a giant virus that infects a marine phagocytic protist [10]. Using metaproteogenomic analysis, another virophage was detected in a sample of a hypersaline meromictic lake in Antarctica; this virophage is found in association with Phycodnaviridae, a giant virus that infects algae [11]. The concept of virophages, small viruses that infect giant virus factories and lead to population control of their hosts, is controversial [12]–[14]. However, this emerging field justifies the exploration of the spectrum of virophages in possible hosts and the design of new tools to complete their repertoire.

The first aim of our study was to investigate the replication capacity of Sputnik 1 and Sputnik 2 in an *A. polyphaga* co-culture with each APM in our collection using quantitative real-time PCR and, more notably, to investigate if virophages previously isolated with group A APM can infect group B or group C viruses. Noting the inability to co-culture a group C virus with its virophage that was detectable by PCR, we decided to inoculate the sample on amoeba and Mimivirus to recover the virophage.

## Materials and Methods

### APM and virophage production

Each virus in our collection (Table 1) was produced by the inoculation of 10 ml of PYG medium with 1 ml each of the individual viral suspension calibrated at 10^6^ particles/ml and the *Acanthamoeba polyphaga* amoebal suspension at 5×10^5^ cells/ml (strain Linc AP-1). After the complete lysis of the amoebas, each co-culture was centrifuged at 2000 rpm for 10 min to pellet the remaining fragments. The supernatant was filtrated through a 0.8 µm pore filter to remove residual amoebas and cysts. The supernatants were frozen at −80°C before being used for viral co-culture inoculation.

**Table 1:** The list of giant viruses and their classification into genotype groups.

Name	Group	GenBank
APM	A	HQ336222
Mamavirus	A	JF979171
Lentille	A	JF979182
Fauteuil	A	JF979168
Pointerouge1	A	JF979167
Cher	A	JF979166
Longchamps	A	JF979169
Terra2	A	GU265562
Lactour	A	JF979173
Courdo5	A	JF979179
Pointerouge2	A	JF979161
Monve	B	JF979181
Moumou	B	GU265560
Terra1	C	GU265563
Courdo7	C	JF979172
Courdo11	C	GU265561
Bus	C	JF979178
Montpellier	C	JF979165
Megavirus chilensis^*^	C	NC_016072

The giant virus labeled with ^*^ is not part of our collection.

Sputnik 1 [8] and Sputnik 2 [9] were produced in co-culture with their natural hosts, Mamavirus and Lentille virus, respectively, in 10 ml of PYG medium containing 1 ml of the amoeba suspension at 5×10^5^ cells/ml, 1 ml of giant virus suspension and 1 ml of each Sputnik strain. After the complete lysis of the amoeba, the purification was performed as described above, but the filtration was executed on three successive filters of 0.8-, 0.45- and 0.22 µm pore sizes to obtain a pure Sputnik suspension. This suspension was prepared before each inoculation assay. These tests were performed in triplicate.

### Sputnik co-culture with APM collection

Each giant virus was inoculated separately into 10 ml of PYG medium containing 1 ml of amoeba suspension at 5×10^5^ cells/ml and 1 ml of each Sputnik suspension. After 1 hour of incubation at 32°C, the supernatant was gently removed to eliminate the Sputnik particles that were not internalized by the amoebas, and 10 ml of fresh PYG medium was added. This time point was defined as H0. The Lentille virus, naturally infected by Sputnik 2, was used as a positive control.

### Real-time PCR assay for evaluation of Sputnik multiplication

After incubation, 200 µl of each co-culture was sampled for DNA extraction and real-time PCR at H1, H0 and Day 3 (to observe the complete lysis of amoebas). The DNA extraction was performed with a Qiamp DNA extraction kit (Qiagen, Hilden, Germany) according to the manufacturer's instructions. Real-time PCR was performed with the LightCycler 480 SYBR Green I Master (Roche) according to the manufacturer's instructions. Sputnik was detected and quantified by using a primer pair targeting the ORF20 of *Sputnik* encoding the major capsid protein (Forward primer 5′-GAGATGCTGATGGAGCCAAT-3′, Reverse primer 5′-CATCCCACAAGAAAGGAGGA-3′). For each sample, the cycle threshold (Ct) was correlated to a reference scale to allow the evaluation of the Sputnik concentration. A total of 200 µl of each co-culture was analyzed by real-time PCR directly after inoculation to assess the quantities of Sputnik 1 and Sputnik 2.

### Microscopic observations

After incubation, 200 µl of each co-culture at H0 and H6 was added to a Cytospin single chamber (Thermo), cytocentrifuged at 800 rpm for 10 min and fixed with methanol. Indirect immunofluorescence microscopy was performed with mouse anti-Sputnik serum as described previously. To label the virus factories and the cell nucleus, we used 5 µl of DAPI (4′, 6′-diamino-2-phenylindole, Molecular Probes). The viable trophozoites were counted using trypan blue (Oxoid) to assess the speed of the lysis of the amoebas by each virus. The preparation of selected samples for transmission electron microscopy was performed as described previously [15] to confirm the results of the quantitative PCR and immunofluorescence assays.

### Pol B gene sequence and phylogeny

The complete Pol B gene sequences for all the viruses used in this study were available from unfinished APM genome sequencing in progress in our laboratory. The sequences of DNA polymerase B were aligned using MUSCLE and trimmed by TrimAL after visual editing. The phylogenetic tree was built with a maximum likelihood using Phyml software.

### Environmental detection of APM and virophages

Tentative detection of APM and virophages was performed on 90 soil samples collected in Marseille and the surrounding areas (south of France). To each 15 g sample, 150 mL of sterile water was added. Decantation occurred overnight at 4°C, and then the samples were filtered, first through Wattman's paper and then through 0.1 µm membrane. Membranes were then put in 1 ml of Page's Amoeba Saline buffer (PAS) and vortexed. The membranes were removed, and the suspension was kept at −80°C prior to use. DNA was extracted with the QIAGEN© QIAmp Mini Kit following the manufacturer's protocol. A combination of several primer pairs was required because the low polB gene conservation in the available fragment [9] prevented the design of broad range primers (Table 2). We have defined the viruses belonging to the Mimivirus group as group A, the viruses belonging to Moumouvirus as group B and the viruses belonging to Courdo11 as group C. Group C includes the recently described *Megavirus chilensis* [6]. Sputnik was detected using the primer pair targeting the ORF20 of *Sputnik*, which encodes the major capsid protein described above.

**Table 2:** Sequences and Tm of the different primers pairs used in the study to amplify partial polB gene of *Mimiviridae*, of group A (Mimi-TJA 01, Mimi-TJA 02), group B (CE11-TE1 01, CE11-TE1 02) and group C (VA10 01).

Primers pair	Sequence	Tm
Mimi-TJA 01	F 5′-GCAGCCCTTTGACACTT-3′	52°C
	R 5′-CATGCGGGAGTTGGAGA-3′	
Mimi-TJA 02	F 5′-GAAAATGGTGAAGAGAAAACTGA-3′	50°C
	R 5′-ACCAGGATAAATGGATGCAA-3′	
CE11-TE1 01	F 5′-AGTTACCCAACCACAAGAAGA-3′	45°C
	R 5′-CAGAAGGACTAACAAAAGAACCA-3′	
CE11-TE1 02	F 5′-AAAATATTGGGGACGTTGGTG-3′	45°C
	R 5′-ATGGAAGACTGGCTGTTGAAA-3′	
VA10 01	F 5′-AAGGGGACAAGGAGTTAAAATAT-3′	45°C
	R 5′-TAGATATACGTTTGGTTTTGGAGTGA-3′	

### Tentative isolation of virophages

To date, virophage cultures have always been obtained by co-isolation with their viral hosts. However, in our study, all attempts to isolate the giant viruses with their virophages in the 5 samples that tested positive using amoeba co-cultures were unsuccessful. Therefore, we designed a new protocol to isolate virophages by inoculating samples on a co-culture of *Acanthamoeba polyphaga* and Mamavirus (used as a reporter virus) [8]. We performed daily blind subcultures and quantitative PCR to detect growth. The five samples detected as positive by PCR were inoculated on 1 mL of PYG with 100 µL of fresh *A. polyphaga* amoebae at a concentration of 5×10^5^ cells/mL and 100 µL of Mamavirus (previously cured of its virophage) at a concentration of 10^6^ pfu/mL. The Mamavirus was confirmed free of virophages by PCR amplification (using the primers described above) before inoculation. Every day, 750 µL of the suspension was mixed with fresh *A. polyphaga* and Mamavirus at the same concentrations. Putative growth of virophages was monitored daily by quantitative PCR.

### Lysis plaque assay with Mimivirus and Sputnik3

Non-nutritive plates containing PAS with 1.5% Agar and 5% of Merck© Hemacolor® solution 3 in square Petri dishes of approximately 520 cm^2^ were used. These plates were inoculated with 30 mL of fresh *A. polyphaga* (5×10^6^ cells/mL) that was previously rinsed in PAS. After one hour of sedimentation, the liquid and surplus of amoeba were removed. Before inoculation, rinsed Mimivirus was suspended in PAS and quantified at 10^9^ infectious particles using an end-point dilution assay. The Sputnik3 aliquots used for inoculation were diluted in PAS and previously filtered through a 0.2 µm pore-sized filter. Sputnik3 was quantified by inoculation of 50 µL of dilutions ranging from the original tube to 10^−15^ in 500 µL of fresh *A. polyphaga* (5×10^5^ cells/mL) rinsed in PAS, infected by Mimivirus (50 µL at 10^9^ pfu/mL). Five daily subcultures were performed in Mimivirus-infected *A. polyphaga*, then 7.5 µL of each well were mixed with 7.5 µL of Mimivirus at 10^9^ pfu/mL and dropped off on plates. Observations three days after the deposits showed smaller diameters of the lysis plaque formations of the originally Sputnik3 not diluted to the 10^−4^ dilution, and almost the same diameters for the other dilutions of Sputnik3 and the Mimivirus-PAS controls: according to these results, we estimated the concentration of Sputnik3 at 10^4^ pfu/mL. Sputnik3 was quantified at 10^9^ particules by mL using a virus-titer estimation protocol with negative staining observed with electron microscopy. Fifteen-microliter deposits were made on amoeba plates, with 25 deposits per plate. Thirty-four deposits were made with 7.5 µL of *Mimivirus* at 10^9^ pfu/mL and 7.5 µL of PAS, 34 with 7.5 µL of *Mimivirus* at 10^9^ pfu/mL and 7.5 µL of Sputnik3 suspension. Deposits of PAS-only and Sputnik3-only were used as negative controls. The plates were checked every day for lysis plaque formation. As the plaques were circular, diameters were measured to calculate mean areas. Statistical comparison was performed by Student's t-Test, using R software (package *stats* version 2.15.0).

## Results

### Pol B gene sequence and phylogeny

**Figure 1:**
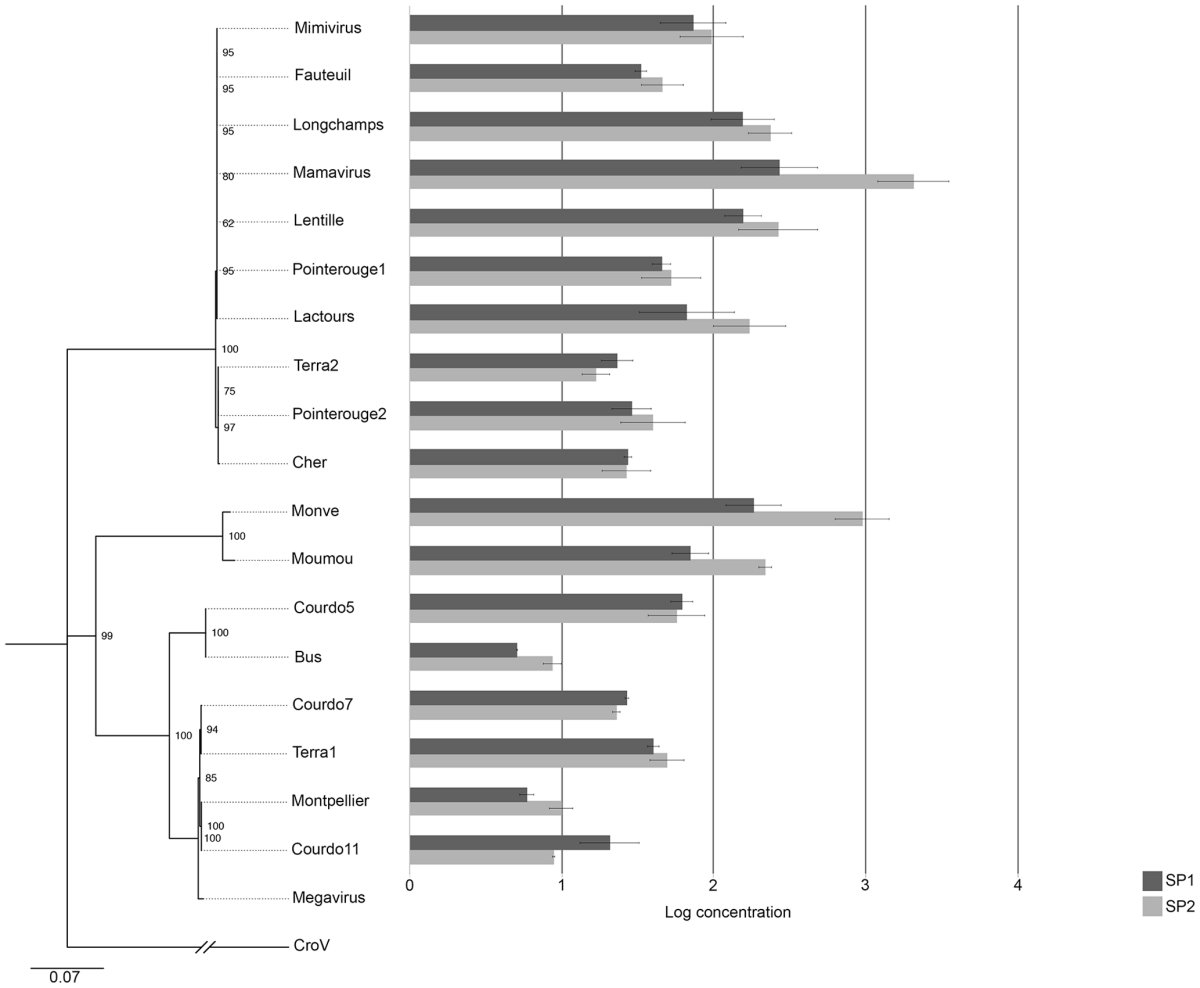
Sputnik 1 and 2 growth in different giant viruses. A histogram of Sputnik 1 and 2 growth in different giant viruses according to their phylogenetic position in the groups A, B and C of Mimiviridae based on Pol B gene sequences. The growth, measured by real-time PCR quantification, was calculated between day 0 and day 3 and corresponded with complete amoebal lysis.

The sequences of pol B gene of all the giant viruses available in our collection (Table 1) were compared with each other. At the protein level, these sequences were identical. At the nucleotide level, the phylogenetic tree, which was built according to the full, 5-kbp sequences of the viral pol B genes, shows a repartition into three groups (Figure 1). The majority of the giant viruses in our collection are clustered in group A.

### Sputnik growth in the 3 groups of Mimiviridae

Each of the 17 Mimiviridae in our collection (10 in group A, 2 in group B and 5 in group C) was inoculated with Sputnik. Using real-time PCR quantification, we demonstrated a 10- to 30-fold increase in Sputnik concentration in most viruses (Figure 1). However, in 2 group C viruses (Bus and Montpellier), we observed only a 5- to 10-fold increase in Sputnik concentration.

**Figure 2:**
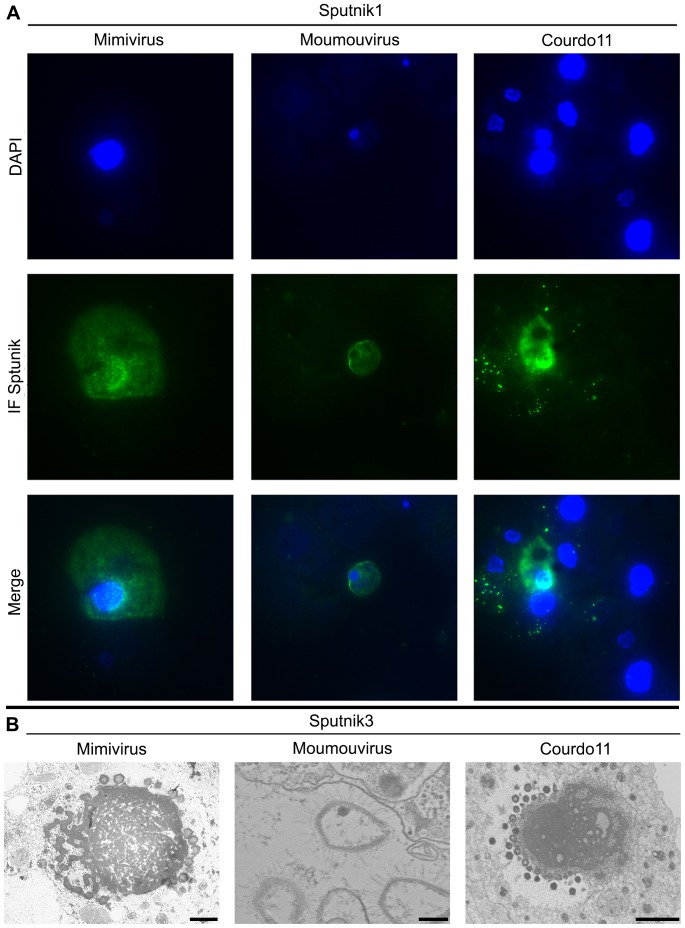
Sputnik within viruses of the different groups of Mimiviridae. (**A**) A DAPI and immunofluorescence labeling of Sputnik 1 within viruses of the groups A (Mimivirus), B (Moumouvirus) and C (Courdo11) of Mimiviridae at 6 h post infection. This figure shows the Sputnik particles labeled with mouse antibody serum (green), and the nucleic acids are indicated by DAPI stain (blue/purple). The virus factories are especially visible by the abundant green stain. (**B**) Aspect of Sputnik3 virophage produced in virus factory of group A Mimivirus (on the left; scale bar 1 µm), group B Moumouvirus (at the center; scale bar 200 nm) and group C Courdo11 (on the right; scale bar 2 µm).

Immunofluorescence observations using Sputnik antibodies and DAPI confirmed the results obtained with real-time PCR. We could observe clearly the viral factories (VF) and the production of Sputnik virions at one pole of the VF (Figure 2) as previously described [15]. For Bus and Montpellier, we observed a weak, diffuse staining of Sputnik (unpublished data) and fewer amoebas (Figure S1). For these two viruses, the number of amoebas declined significantly and more rapidly between H15 and H18 compared with the 15 other isolates, whether co-infected with Sputnik or not, and reached levels similar to those observed between H36 and H40 with the viruses of the APM group (Figure S1). The production of Sputnik by these two isolates was checked by TEM at 6 and 12 h post-infection, but no Sputnik virions were observed. This result is consistent with the diffuse staining observed using immunofluorescence. The more rapid multiplication of these strains may explain the lower Sputnik production.

### Detection of Mimiviridae and virophages in the environment

We tested 90 soil samples collected in Marseille and surrounding areas (south of France) for Mimiviridae and virophage using PCR. We detected 9 Mimiviridae: 8 group A strains and 1 group C strain. We detected 5 virophage sequences, one of which was associated with the group C virus mentioned above. Detection of the other 4 virophages was not associated with the detection of a giant virus. The available partial sequence together with both the sequences of the giant viruses that we studied previously and the recently described *Megavirus chilensis* was used to build a phylogenetic tree (Figure S2) [6], [9]. Our efforts to grow the group C virus potentially associated with a virophage failed, and we concluded that our amoeba support was ineffective. Therefore, we tried to isolate the virophage alone on a co-culture of amoeba and Mamavirus.

### Isolation of virophage by co-culture with Mamavirus and amoebae

**Figure 3:**
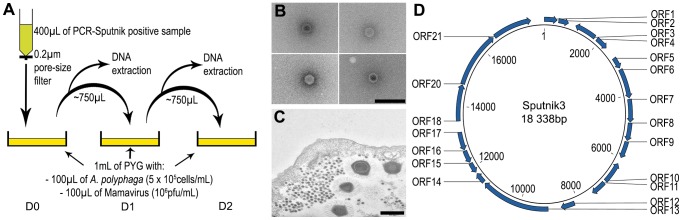
Sputnik 3 virophage isolation. (**A**) Schematic summarizing the protocol used in this study for the isolation of virophage. Observations of the virophage isolated in this study by (**B**) transmission electron microscopy of negatively stained particles (A, scale bar 200 nm) and (**C**) transmission electron microscopy of thin section of culture (B, scale bar 500 nm). (**D**) Genome of Sputnik 3.

Prepared soil samples were inoculated on the co-culture, and we performed daily blind subcultures and quantitative PCR to detect growth (Figure 3a). Of the 5 samples in which a virophage was detected after 5 blind subcultures, only the soil sample containing the group C virus sequence demonstrated increasing concentration of virophage DNA as determined by real-time quantitative PCR (data not shown). This was confirmed by electron microscopy (Figure 3 b and c). As soon as growth of this potential virophage was detected, production was performed on *A. polyphaga* newly infected by Mamavirus in PYG (1∶1∶10 ratio in volume, respectively). Daily subculture was performed at a 1∶2 ratio (a volume of culture in the double of fresh amoeba infected by Mamavirus). Later, we were able to propagate this virophage on Courdo11, a group C strain, and on Moumouvirus, a group B strain. The complete genome sequencing of Sputnik3 (GenBank accession number: JN603370) allowed a comparison with the genomes of virophages Sputnik (GenBank: EU606015) and Sputnik2 (GenBank: JN603369) (Figure 3d and Figure S3). Sputnik2 and Sputnik3 show four differences compared with the first Sputnik identified: insertions-deletions (in-dels) of an adenine base at positions 877 and 7949/7951 and in-dels of a thymine base at positions 12936 and14047 (the Sputnik genome was used as the reference for nucleotide numbers). The adenine in-dels are located between ORFs and therefore do not affect the coding sequence. However, the first in-del of a thymine is located in gp17, the gene encoding a putative IS3 family transposase A protein; the change causes a shift in the reading frame and is associated with an extended gp17 in Sputnik2 and Sputnik3 (88 amino acids in Sputnik, and 187 in Sputnik2 and Sputnik3) (Figure S4). The second in-del of a thymine occurs at a crossing point between gp18 and gp19 and results in a single ORF instead of gp18 and gp19. However this appeared to be actually an initial mistake from the sequencing of Sputnik as recently proved (Zhang *et al*, 2012 [16]), and thus gp18 and gp19 are Sputnik3 has three substitutions compared with Sputnik and Sputnik2. The first substitution, adenine to thymine, is located between gp12 and gp13 at position 8986/8991. A guanine to adenine change is located at position 16098 in ORF20, which encodes the putative major capsid protein. This substitution changes a nonpolar alanine to a polar threonine and could affect the function of the putative protein or its structure, since the mutation occurs in a predicted beta strand. The last substitution, a guanine to adenine change at position 17666 in ORF21, induces a change from an aspartic acid to the polar amino acid asparagine, potentially affecting the putative function of the unknown protein encoded by this ORF. The ORF20 of Sputnik 3 also differed by 3 nucleotides compared to that obtained directly by PCR on the soil sample.

### Suspicion that Sputnik3 virophage is a pathogen for Mimivirus

Within the first day after inoculation, cythopathogenic effects associated with Mimivirus lysis on amoeba appeared on culture plates as circular clear areas or lysing plaques. The plaques were almost identical for all deposits (Sputnik3-infected Mimivirus and control Mimivirus with PAS) and extended until the third day (Figure S5a–b). No plaques appeared on negative controls (PAS-only and Sputnik3-only), even after 7 days. The plaque surfaces for the control Mimivirus-PAS deposits ranged from 0.63 cm^2^ to 1.21 cm^2^ with a mean area of 0.88 cm^2^ (±0.15 cm^2^), whereas the plaques for the Sputnik3-infected Mimivirus deposits ranged from 0.22 cm^2^ to 0.72 cm^2^ with a mean of 0.56 cm^2^ (±0.12 cm^2^) (Figure S5c). These differences were significant according to the Student's t-Test (p-value 4.49e^−15^). Cross dilutions of Mimivirus and Sputnik3 revealed that plaque size was not dependent on Sputnik3 concentration, but the deposits with 10^5^ pfu/mL of Mimivirus plaques were smaller and difficult to measure. The effect of Sputnik3 on Mimivirus was confirmed by real-time PCR on co-culture (Figure S6).

## Discussion

Our study shows that Sputnik 1 and Sputnik 2 are able to replicate in all of the APM virus factories. Immunofluorescence analyses confirmed the polar production of Sputnik viruses in the periphery of the giant virus factory as described previously [15]. The quantitative PCR results confirm these observations, although we observed a lower multiplication in the group C viruses Montpellier and Bus. However, virophages multiplied efficiently in the group C viruses Courdo7, Courdo 11 and Terra1. We hypothesized that the small Sputnik viral particles, trapped between the surrounding fibers, are phagocytosed along with the giant virus, as observed previously [15] because amoebas have the capacity to phagocytize particles of a size greater than 0.5 µm, including latex beads [17]. In a recent study, we observed that after 150 passages in its amoeba host, the genome of APM shifted dramatically from 1.2 to 0.993 Mbp and was associated with deletions of genes encoding two proteins (R135 and L725) associated with wild-type virus fibrils. This APM clone lacking surrounding fibrils was not able to propagate the Sputnik virophages [18]. Using TEM, Montpellier and Bus were observed to have fibrils comparable to that of other APMs, including other group C APMs [9]. Thus, a difference in fibrils is not responsible for the lower level of multiplication. The lack of efficient virophage multiplication in Montpellier and Bus seems associated with the rapid amoeba lysis observed with these viruses, which leads to limited multiplication. The observed diffuse immunofluorescence may reflect the production of sputnik proteins without the formation of mature particles.

The isolation of an additional virophage, Sputnik 3, using an original procedure confirms the capability of virophages to be cultured in viruses of the 3 groups of APM. We speculated that we were not able to isolate the virus detected in the sample from which we isolated the virophage because the amoeba we used was not susceptible to this virus [19]. We were able to propagate this virophage on strains of group B and C viruses, demonstrating that Sputnik 3 is able to infect strains from all 3 groups of *Mimiviridae*. Sputnik 3 is the first virophage recovered from group C, which contains *Megavirus chilensis*. The growth of Sputnik 3 in Moumouvirus, the third group of Mimiviruses, further demonstrates that, like Sputnik 1 and 2, Sputnik 3 is able to infect the 3 groups of Mimivirus. This result supports the hypothesis of the ability of virophages to drive gene transfer, potentially contributing to the mosaicism of giant viruses' genomes. This fuels the debate on the real nature of virophages[14]. The highly suspected deleterious effect of sputnik 3 on amoeba lysis associated with Mimivirus infection (Figures S5 and S6) argues against the theory of a simple satellite. Our data suggest that the host range of virophages is wider than previously thought.

Finally, the procedure reported here, which uses a cultivable helper giant virus, paves the way for the isolation and discovery of new virophages without the isolation of their giant virus host. Isolating virophages using a reporter giant virus is significant because diverse virophage signatures have been identified in nearly all types of aquatic systems by searching the GOS data base and thus are likely to play a key role in the ecosystem regulation of aquatic environments by regulating host-virus interactions [15]. Isolating other virophages using a Phycodnaviridae-algae co-culture to grow those detected by metagenomic analysis only [11] will facilitate our understanding of their biology, including their developmental cycle and host specificity.

## Supporting Information

Figure S1 **Number of amoebas in PYG medium.** **A**: The number of viable amoebas present during the infection by giant viruses. **B**: The number of viable amoebas present during co-infection with giant viruses and Sputnik 1. **C**: The number of axenic amoebas compared with the number of amoeba infected by Mamavirus (Green: Montpellier, Blue: Bus, Orange: Courdo 5, Red: Pointerouge 1, Purple: Mamavirus, Black: control uninfected amoeba). Statistical analysis was performed according to the Wilcoxon Rank Sum test performed with R software using the package *stats* version 2.15.0 5 [Hollander, M. & Wolfe, D. A. (1973) *Nonparametric Statistical Methods* (John Wiley and Sons). Bauer, F. B. (1972) *Journal of the American Statistical Association* 67, 27–33].
(TIF)

Figure S2 **Position of giant viruses detected in the soil compared to currently known Mimiviridae.** Phylogenetic trees based on partial polB gene sequence showing the position of giant viruses detected in the soil (T17,T16,T12, T32, T39, T71, C5 and C6) compared to currently known members of the Mimiviridae family presented in our former study [La Scola B, et al. (2010) Tentative characterization of new environmental giant viruses by MALDI-TOF mass spectrometry. *Intervirology* 53:344–53].
(TIF)

Figure S3 **Alignment of the genomes of the 3 isolates of Sputnik.**
(TIF)

Figure S4 **Sequences of ORF17 (Gp17), ORF20 (Gp20) and ORF21 (Gp21) in Sputnik, Sputnik2 and Sputnik3.** The black line indicates that the sequence is the same that the one just above. The amino acids in red are those that are different between the three Sputnik. In the Gp21 sequence, for which predicted secondary structures are available (RCSB Protein Data Bank at www.rcsb.org, ID: 3J26; [16]), green boxes indicate beta strand and blue boxes indicate alpha helix.
(TIF)

Figure S5 **Lysis plaque assay with Mimivirus and Sputnik3.** (**A**) Scan of a colored lysis plaques with *A polyphaga* monolayer inoculated with Mimivirus (4 right spots, 3 to 6) and Mimivirus and Sputnik3 (2 left spots, 1 and 2) 3 days after inoculation; (**B**) magnification of a right spot; (**C**) difference of lysis plaques means measured on colored plates 3 days following inoculation, between 34 deposits of *Mimivirus* and 34 deposits of Mimivirus and Sputnik (Spt3).
(TIF)

Figure S6 **Quantification of Mimivirus and amoebal lysis.** Quantification of Mimivirus by real-time PCR (curves), with Sputnik3 (red triangle) and without (blue square) from H0 to H24 post-infection, from co-culture in *Acanthamoeba polyphaga* in PAS (non-infected amoebas were used as negative control and provided no amplification). The bars represent the number of amoebas for each time: Mimivirus-infected amoebas in blue, Mimivirus/Sputnik3-infected amoebas in red and non-infected amoebas in green.
(TIF)
