# Supplementary figures and images for: Broad Spectrum of Mimiviridae Virophage Allows Its Isolation Using a Mimivirus Reporter
Source: PLoS One. 2013 Apr 15;8(4):e61912. doi: 10.1371/journal.pone.0061912 (PMC3626643; doi:10.1371/journal.pone.0061912)

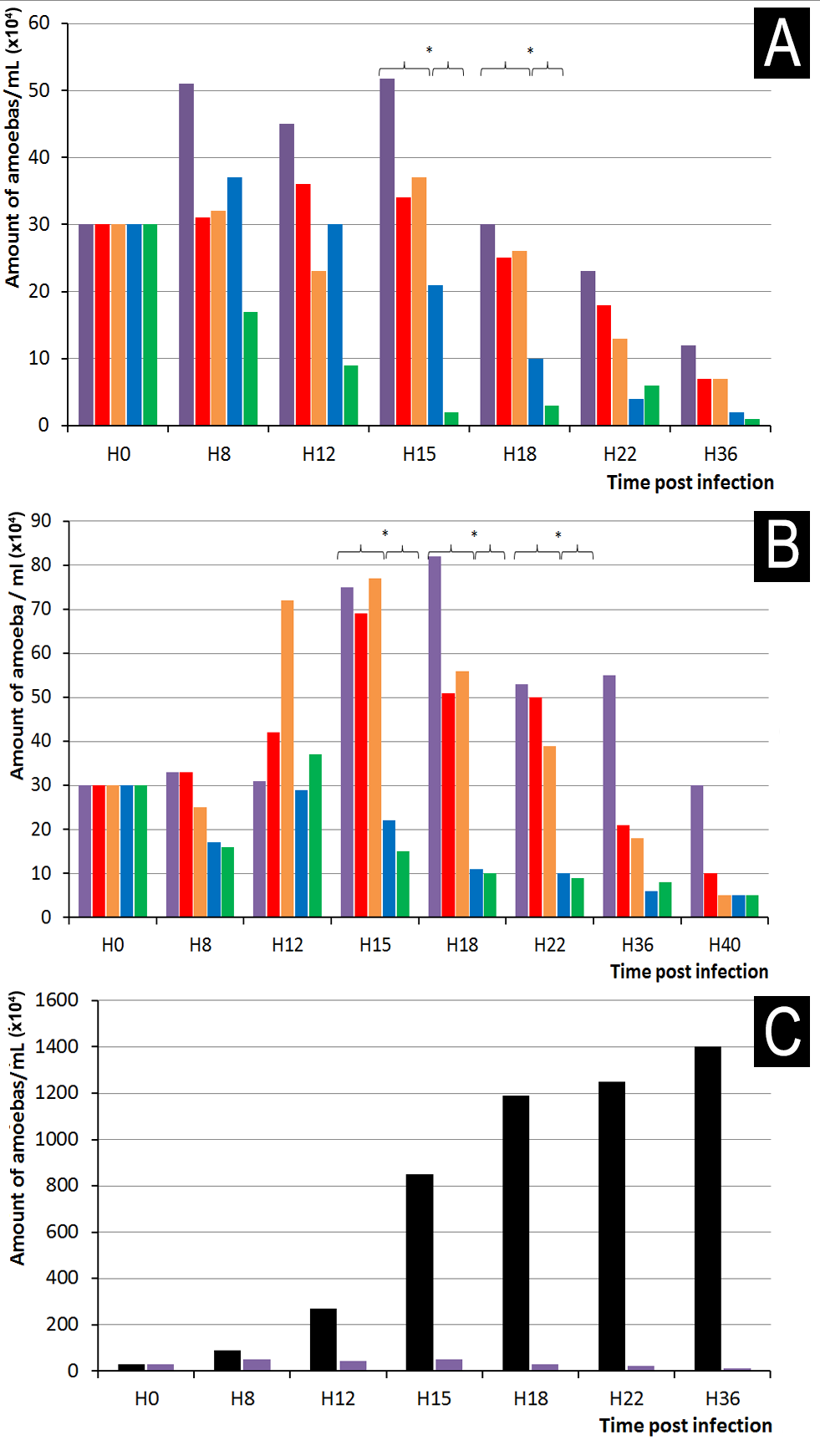

Supplement: Figure S1 — Number of amoebas in PYG medium. A: The number of viable amoebas present during the infection by giant viruses. B: The number of viable amoebas present during co-infection with giant viruses and Sputnik 1. C: The number of axenic amoebas compared with the number of amoeba infected by Mamavirus (Green: Montpellier, Blue: Bus, Orange: Courdo 5, Red: Pointerouge 1, Purple: Mamavirus, Black: control uninfected amoeba). Statistical analysis was performed according to the Wilcoxon Rank Sum test performed with R software using the package stats version 2.15.0 5 [Hollander, M. & Wolfe, D. A. (1973) Nonparametric Statistical Methods (John Wiley and Sons). Bauer, F. B. (1972) Journal of the American Statistical Association 67, 27–33]. (TIF) [file pone.0061912.s001.tif]

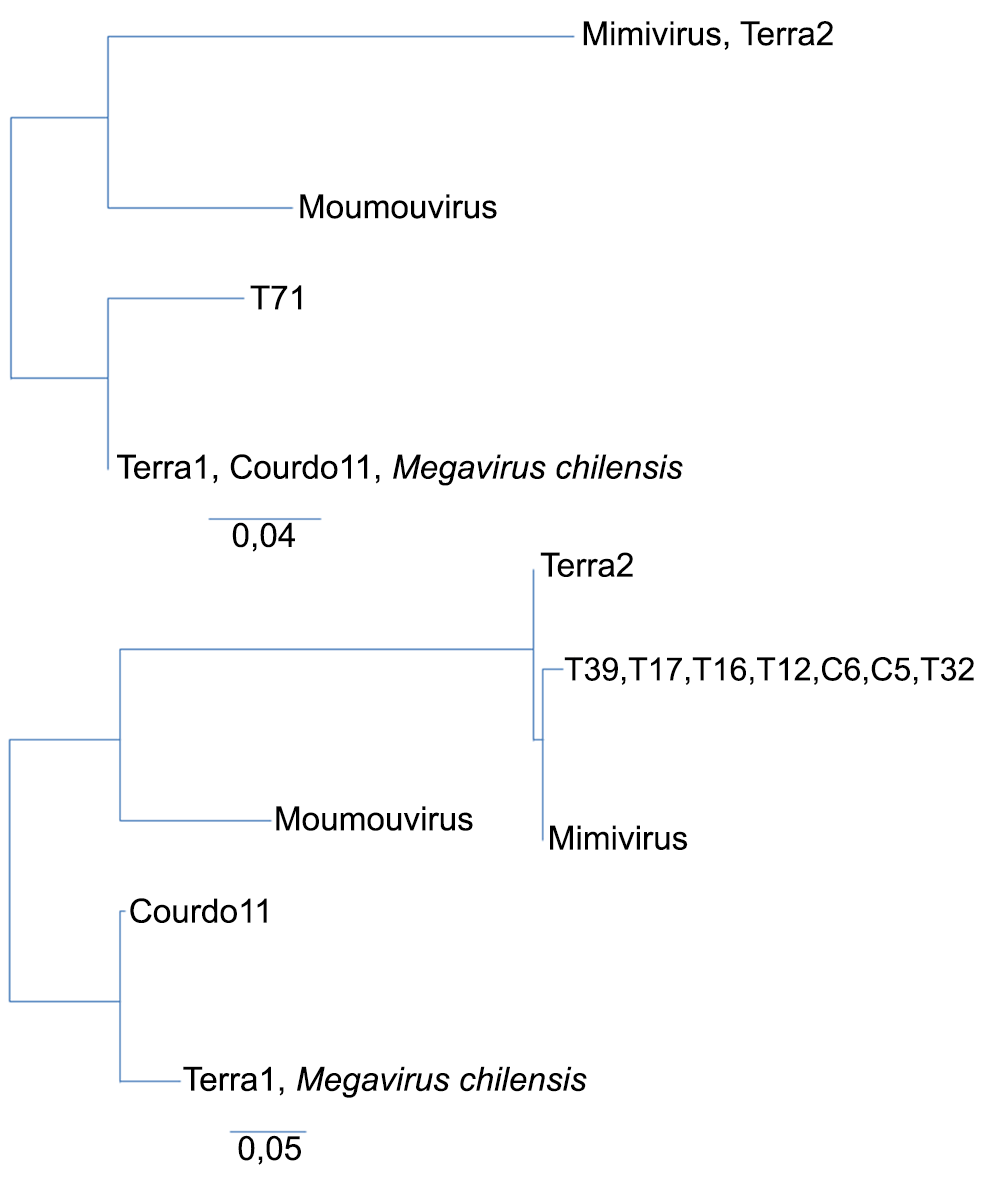

Supplement: Figure S2 — Position of giant viruses detected in the soil compared to currently known Mimiviridae. Phylogenetic trees based on partial polB gene sequence showing the position of giant viruses detected in the soil (T17,T16,T12, T32, T39, T71, C5 and C6) compared to currently known members of the Mimiviridae family presented in our former study [La Scola B, et al. (2010) Tentative characterization of new environmental giant viruses by MALDI-TOF mass spectrometry. Intervirology 53:344–53]. (TIF) [file pone.0061912.s002.tif]

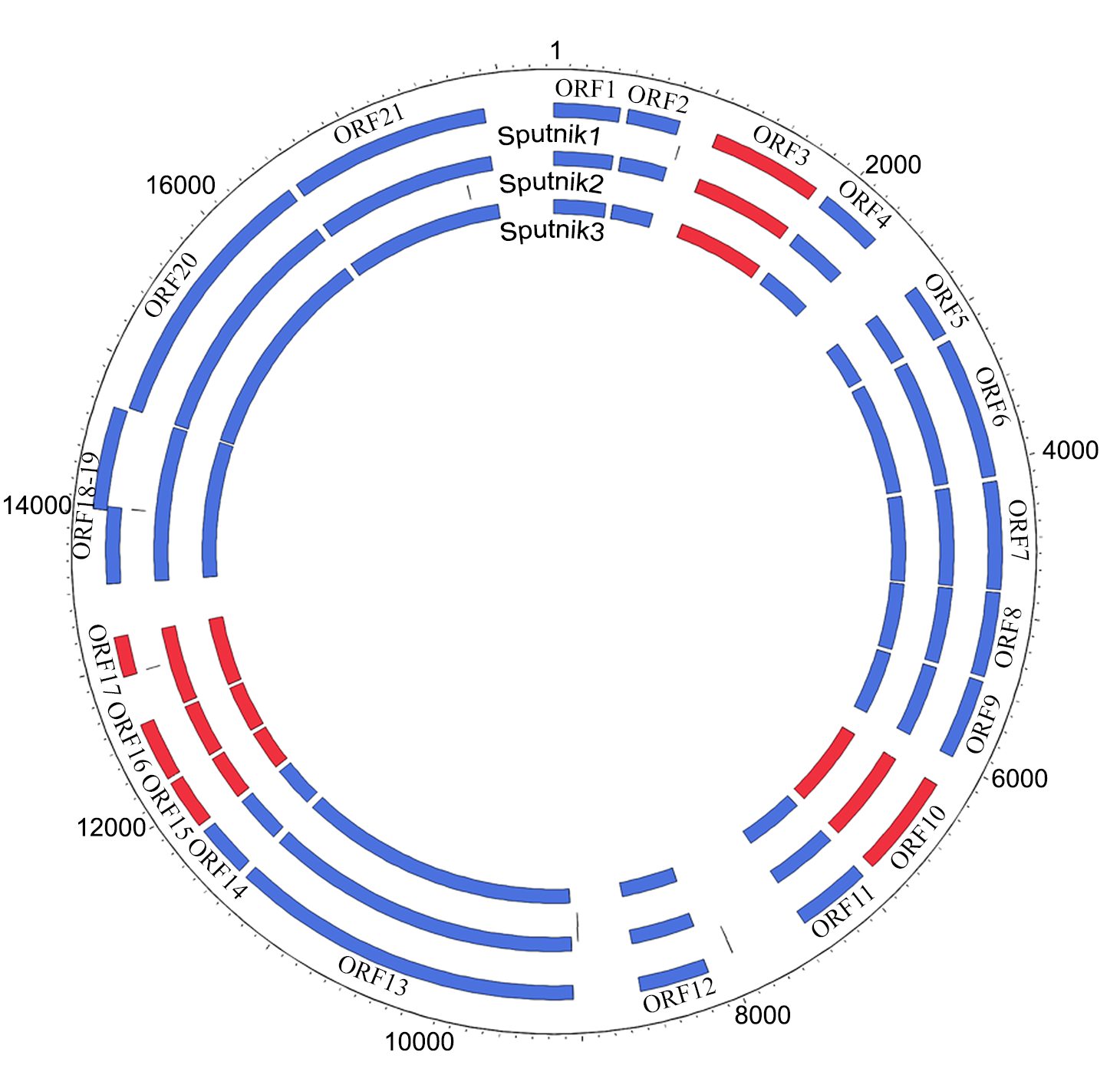

Supplement: Figure S3 — Alignment of the genomes of the 3 isolates of Sputnik. (TIF) [file pone.0061912.s003.tif]

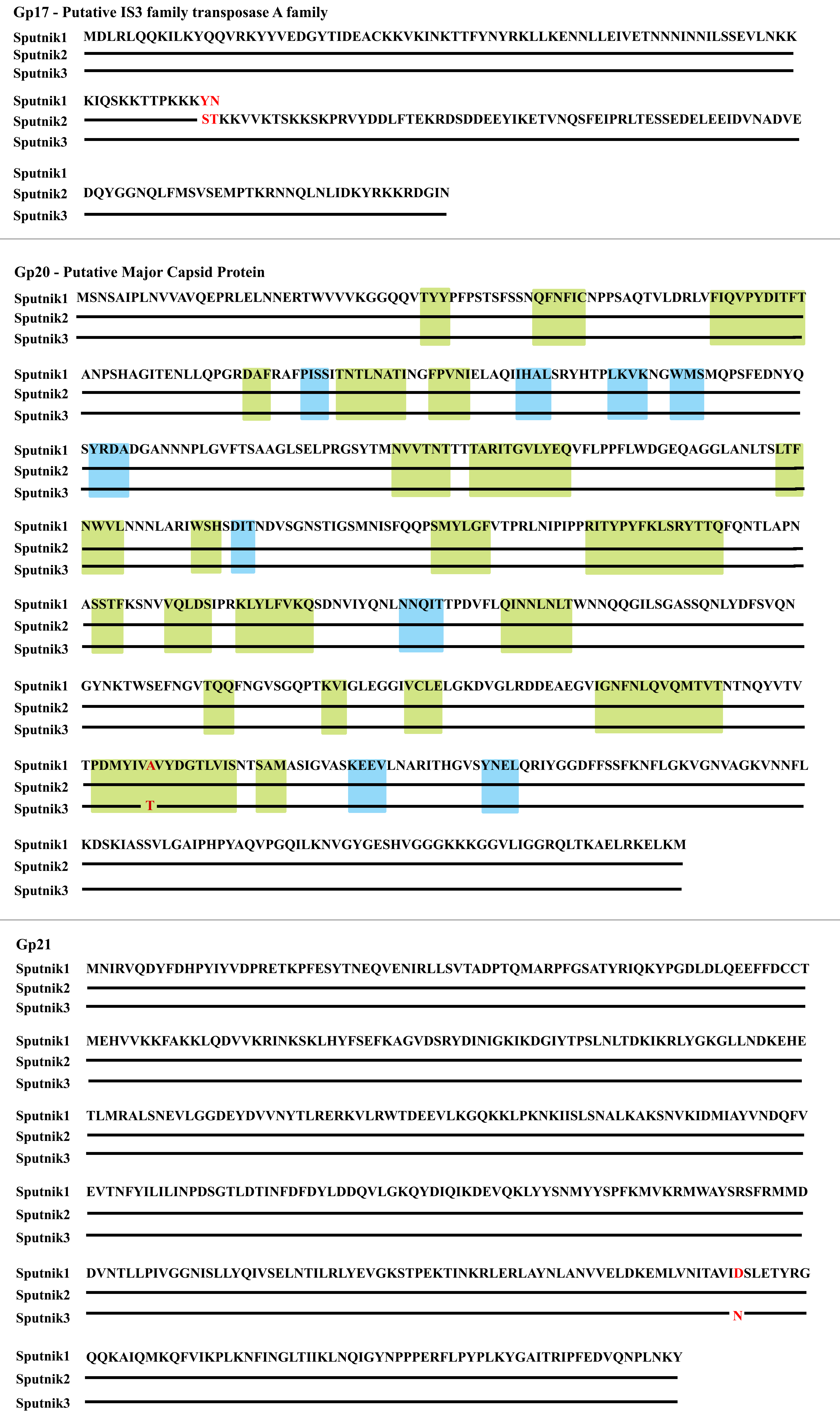

Supplement: Figure S4 — Sequences of ORF17 (Gp17), ORF20 (Gp20) and ORF21 (Gp21) in Sputnik, Sputnik2 and Sputnik3. The black line indicates that the sequence is the same that the one just above. The amino acids in red are those that are different between the three Sputnik. In the Gp21 sequence, for which predicted secondary structures are available (RCSB Protein Data Bank at www.rcsb.org, ID: 3J26; [16]), green boxes indicate beta strand and blue boxes indicate alpha helix. (TIF) [file pone.0061912.s004.tif]

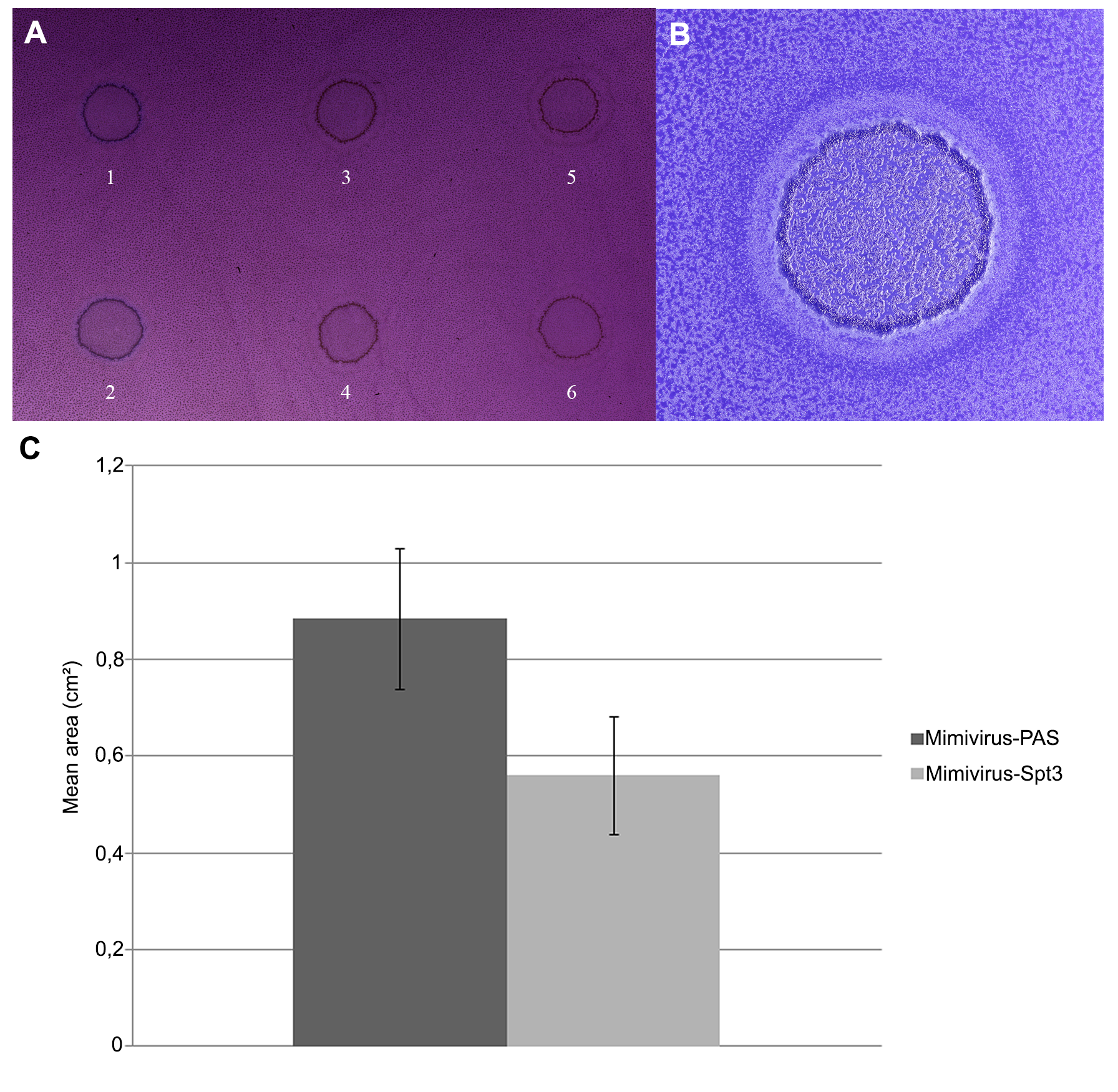

Supplement: Figure S5 — Lysis plaque assay with Mimivirus and Sputnik3. (A) Scan of a colored lysis plaques with A polyphaga monolayer inoculated with Mimivirus (4 right spots, 3 to 6) and Mimivirus and Sputnik3 (2 left spots, 1 and 2) 3 days after inoculation; (B) magnification of a right spot; (C) difference of lysis plaques means measured on colored plates 3 days following inoculation, between 34 deposits of Mimivirus and 34 deposits of Mimivirus and Sputnik (Spt3). (TIF) [file pone.0061912.s005.tif]

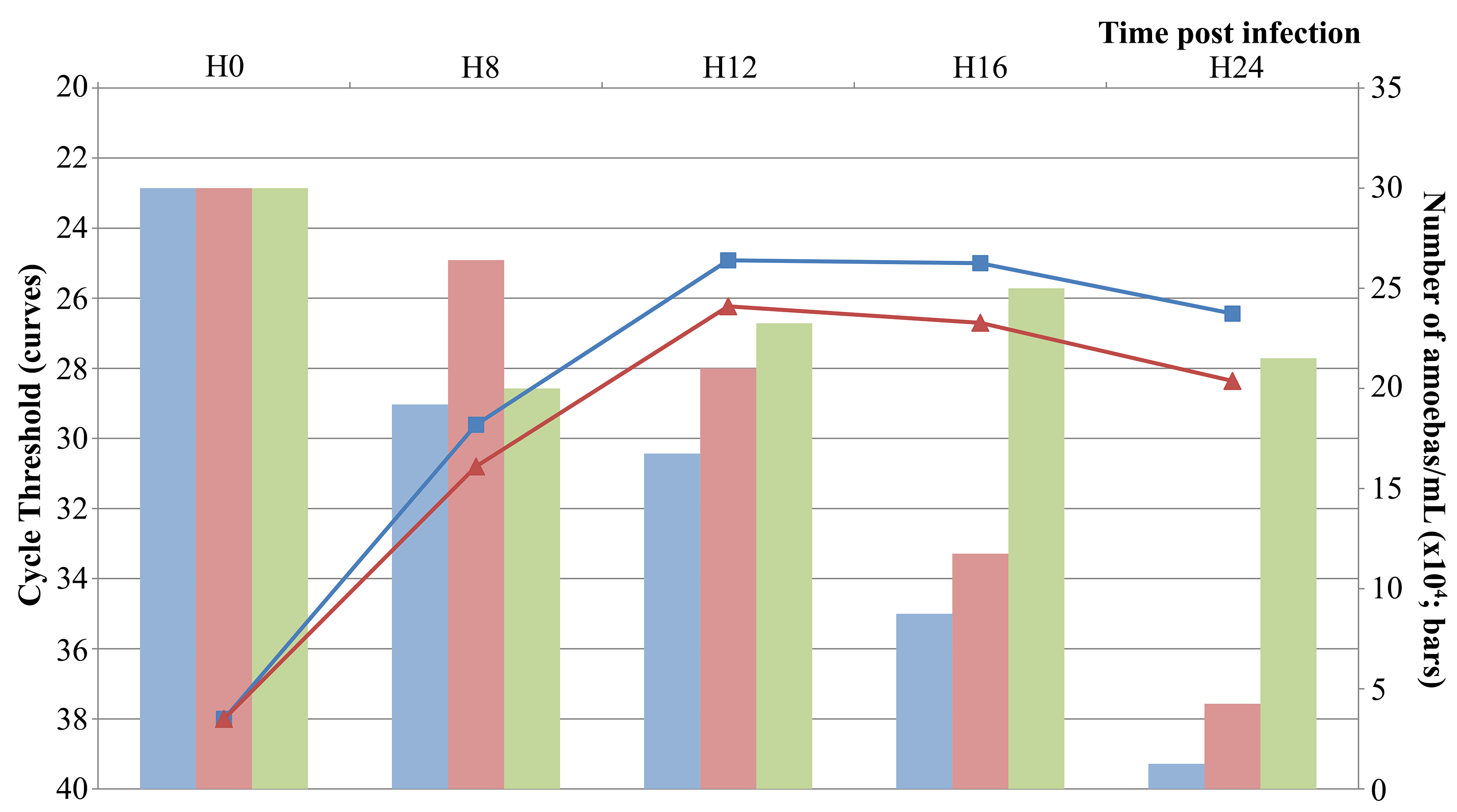

Supplement: Figure S6 — Quantification of Mimivirus and amoebal lysis. Quantification of Mimivirus by real-time PCR (curves), with Sputnik3 (red triangle) and without (blue square) from H0 to H24 post-infection, from co-culture in Acanthamoeba polyphaga in PAS (non-infected amoebas were used as negative control and provided no amplification). The bars represent the number of amoebas for each time: Mimivirus-infected amoebas in blue, Mimivirus/Sputnik3-infected amoebas in red and non-infected amoebas in green. (TIF) [file pone.0061912.s006.tif]
